# Are We Playing It Fast and Loose With the Serofast?

**DOI:** 10.1097/INF.0000000000004585

**Published:** 2025-01-10

**Authors:** Brent D. Nelson, Shelley M. Lawrence, Kelsey Simek, Kimberly B. Stowers, Jose E. Morales Moreno, Jeffrey S. Prince, Sonia Mehra

**Affiliations:** From the * Division of Infectious Diseases; † Division of Neonatology; ‡ Division of General Pediatrics, Department of Pediatrics, University of Utah; § Department of Medical Imaging, Primary Children’s Hospital, Salt Lake City, Utah.

**Keywords:** congenital syphilis, serofast, serologic nonresponse, late latent syphilis, maternal syphilis

## Abstract

Four infants (one singleton and a set of triplets) born to mothers with serofast reactive plasma reagin at 1:4 to 1:8 were found to have congenital syphilis. Each mother had a history of receiving appropriate treatment for their syphilis stage at the time of diagnosis with benzathine penicillin G 2.4 million units intramuscularly weekly 3 times. Both exhibited a 4-fold decrease in their reactive plasma reagin titer. The singleton was asymptomatic but found to have long bone radiographic evidence for congenital syphilis. Of the triplets, one had laboratory abnormalities and a rash, while all 3 triplets had radiographic findings of congenital syphilis. All were treated with 10–14 days of intravenous penicillin G according to the Centers for Disease Control and Prevention treatment guidelines. These cases highlight that infants may be at risk for congenital syphilis, despite being classified as “congenital syphilis unlikely” according to current syphilis practice guidelines. A thorough maternal history and complete infant physical evaluation at birth is recommended for all neonates born to mothers diagnosed with and treated for syphilis. Close follow-up with repeat nontreponemal titers, as outlined by the Centers for Disease Control and Prevention and American Academy of Pediatrics guidelines, is recommended.

Globally, cases of congenital syphilis are rising and represent an alarming trend and public health crisis.^1^ Within the United States, the Centers for Disease Control and Prevention (CDC) guidelines and many state laws mandate timely universal screening for syphilis at the first prenatal visit during the first trimester of pregnancy.^2,3^ Recently, the American College of Obstetricians Gynecologists expanded screening recommendations to include universal screening at the first antenatal visit, third trimester (ie, around 28 weeks of gestation) and at delivery.^4^

Treatment of syphilis during pregnancy is dependent on the stage of infection and mirrors recommendations for nonpregnant individuals.^3^ Adequate response to therapy is determined by clinical and serologic response monitored by nontreponemal lipoidal antigen (LA) testing either via the reactive plasma reagin (RPR) or venereal disease research laboratory test. An appropriate response to therapy is determined by either a 4-fold decrease in LA antibody titers or complete “seroreversion,” sometimes referred to as “serological cure.”^5,6^ As demonstrated in Figure 1, some individuals, however, never “serorevert” and remain “serofast” or “serologic nonresponders” with low, but persistently reactive titers.^6^ Although rare, some patients are observed to have a less than 4-fold decrease in their LA antibody titers, despite adequate treatment.^6^ Recently within our hospital system, we cared for 4 infants (1 individual birth and a set of triplets) born to mothers with a history of syphilis. The mothers had documented appropriate treatment by the local health department, for their stage of syphilis prior to conception, but their RPR remained at low, serofast levels of 1:4 to 1:8. Despite no concern for reinfection with *Treponema pallidum*, these mothers gave birth to infants with clinical findings consistent with congenital syphilis. It is not fully understood why some individuals remain serofast while others demonstrate seroreversion after treatment. This phenomenon is most often observed in individuals who had an initial diagnosis of late latent syphilis or syphilis of unknown duration.^6^ Persons treated during secondary stages of syphilis may also remain serofast, which is thought to be secondary to undetected cases of neurosyphilis or the presence of *T. pallidum* in other host immune privileged sites, such as the anterior chamber of the eye.^7,8^

**FIGURE 1.**
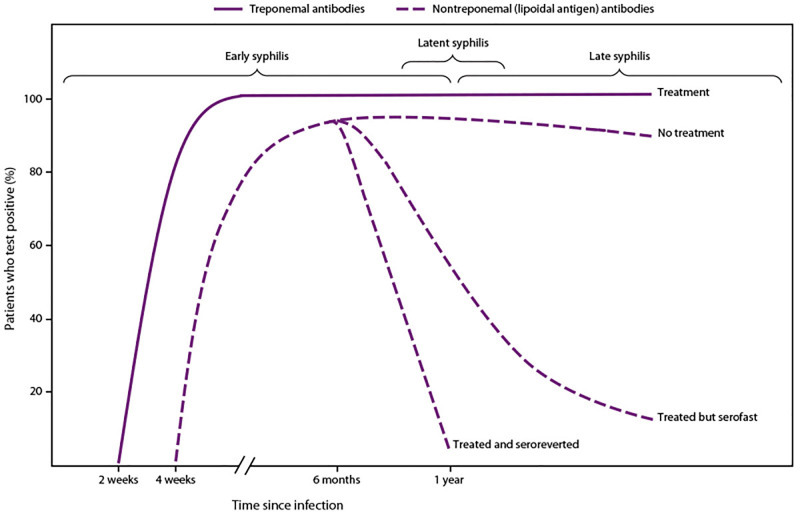
Serologic response to infection with *Treponema pallidum*.

There is also controversy regarding the number of doses of penicillin G required for the treatment of primary, secondary and early latent syphilis in pregnant women. While a single dose of 2.4 million units administered once intramuscularly (IM) had an efficacy of 98% overall for all stages of syphilis, the effectiveness of this regimen was lower (95%) for pregnant women with secondary syphilis.^9^ As a result, treatment failure of the fetus is estimated to occur in between 2% and 14% of pregnancies.^10,11^ Therefore, the CDC treatment guidelines for primary, secondary and early latent syphilis in pregnant women indicate that a second dose of benzathine penicillin G 2.4 million units administered IM 1 week after the initial dose may be useful to prevent fetal treatment failures.^10^

Currently, available syphilis diagnostics are limited, and detection of persistent infection is often difficult. In one observational study by Zhang et al,^12^ patients who were serofast underwent retreatment and cerebrospinal fluid (CSF) analysis with a small percentage (3%–10%) of serofast individuals identified to have neurosyphilis in subsequent follow-up. While the data in this study suggested that serofast patients did not likely benefit from additional therapy in the short term, the long-term impact remains unknown. A separate study by Zhou et al^7^ analyzed 17 cases of neurosyphilis in immunocompetent patients who remained serofast 24 months after appropriate treatment for secondary syphilis. These investigators found improved clinical manifestations of neurologic or psychiatric symptoms in 4, resolution of CSF leukocytosis in 9 and decline of CSF protein in 9 patients. This suggests that there is possibly a small cohort of individuals who have been “adequately treated” according to approved treatment guidelines but may continue to have underlying *T. pallidum* infection. With the presentation of this case series, we hope to illuminate the possibility of vertically transmissible, low-level infection in serologic nonresponders despite “adequate” treatment.

## CASE 1

**FIGURE 2.**
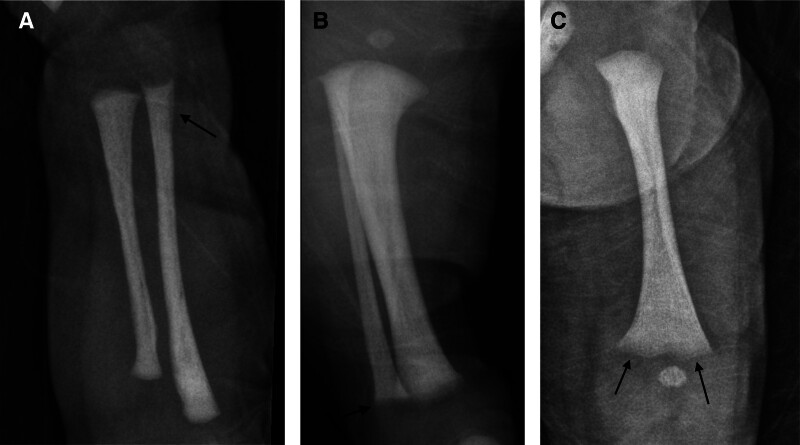
A: Radiograph of the right forearm demonstrates cupping of the distal radial metaphysis as well as periostitis (black arrow). B: Radiograph of the right tibia and fibula demonstrates irregularity of the distal tibial and fibular metaphyses (black arrow). C: Radiograph of the left femur demonstrates loss of mineralization in the distal femoral metaphysis as well as loss of the zone of provisional calcification in the subchondral bone (black arrows).

- Singleton pregnancy, 37 weeks 2 days.
- Nine years prior to delivery, the mother was diagnosed with latent syphilis with a documented RPR titer of 1:32. She received 3 weekly doses of benzathine penicillin G 2.4 million units IM 2 weeks later.
- Follow-up testing was performed at multiple labs with conflicting results. Four and nine days after completion of therapy, titers were 1:32 and 1:256, respectively, at separate locations. A repeat titer 1 month later remained at 1:32 at the public health lab.
- Due to discrepant results, she was retreated with 3 weekly doses of benzathine penicillin G 2.4 million units IM. Both treatment regimens were documented with the state’s public health department.
- Repeat RPR at 2 and 4 months posttreatment was 1:32 and 1:16, respectively, at the public health lab. The same sample at 4 months posttreatment was 1:64 at the non public health lab.
- Seven years prior to delivery and 2 years after recent treatment, the mother’s RPR was reported as 1:64 at both labs. She received a single dose of benzathine penicillin G 2.4 million units IM, whether for concern of treatment failure or reinfection was not clear in the medical record. This treatment was also documented by the state’s public health department.
- Two years prior to delivery, RPR decreased to 1:8 and later 1:4 at the time of delivery.
- Due to unavailability of maternal syphilis treatment records at delivery, a full workup for congenital syphilis was initiated, including a complete blood count, complete metabolic panel, lumbar puncture, long bone radiographs, diagnostic hearing evaluation and dilated eye examination.
- The findings of abnormal long bone radiographs consistent with congenital syphilis including mild cupping of metaphyses and areas of demineralization were detected involving the bilateral distal radii, ulna, femur, tibia and fibula (Fig. 2). The CSF was uninterpretable due to clotting of the specimen. The remaining workup was unremarkable.
- The infant was treated with 10 days of aqueous crystalline penicillin G 50,000 U/kg.
- The infant’s RPR at birth was 1:2 and a follow-up RPR at 2 months of age was nonreactive.

## CASE 2

- The pregnancy was conceived by in vitro fertilization, resulting in a triplet gestation with delivery at 33 weeks 0 days.
- Maternal syphilis was diagnosed and treated 29 years prior to delivery outside of the United States. Her partner was treated at the same time. The couple divorced soon after.
- The mother remarried and her RPR was measured twice: at the time of entry to the United States (6 years prior to current pregnancy) and during previous in vitro fertilization twin pregnancy (3 years prior to current pregnancy). The RPR titer was 1:8 on both measurements and she was treated by the local health department with 3 weekly doses of penicillin G 2.4 million units IM for both occurrences.
- Her RPR was measured during the first trimester of her most recent pregnancy and resulted at 1:8. This pregnancy was conceived using an anonymous egg donor and known sperm donor (her new husband who tested negative for syphilis).
- Two maternal RPR titers obtained 10 and 9 days prior to delivery were 1:2 and 1:4, respectively. With discrepant results, she was restarted on treatment, receiving 1 dose of penicillin 2.4 million units IM before delivery. She completed treatment with 2 more doses of IM penicillin after the birth of her triplets.
- All infants received complete workup for congenital syphilis including complete blood count, complete metabolic panel, lumbar puncture, long bone radiographs, diagnostic hearing evaluation and dilated eye examination.

### Triplet A

**FIGURE 3.**
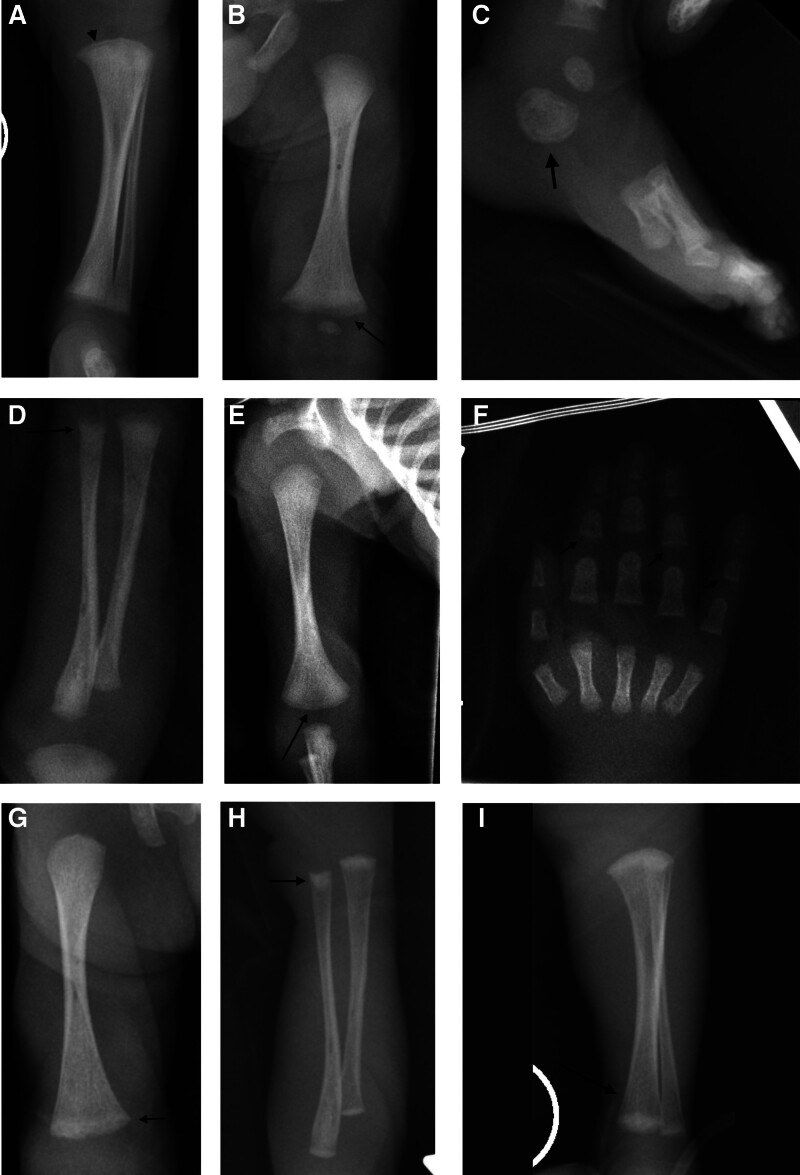
Radiographs (A–C), (D–F) and (G–I) correspond to triplet A, B and C, respectively. A: Radiograph of the left tibia and fibula demonstrates a celery stalk appearance of the distal tibia and fibula (black arrow) and subchondral lucent band in the proximal tibial metaphysis (black arrowhead). B: Radiograph of the left femur demonstrates demineralization of both the proximal and distal femoral metaphyses with irregularity of the distal metaphysis (black arrow). C: Lateral radiograph of the left foot demonstrates double density in the calcaneus (black arrow), which could represent disrupted mineralization associated with congenital syphilis. D: Radiograph of the right forearm demonstrates cupping of the distal ulnar metaphysis with mixed lucency and sclerosis in both the distal radial and ulnar metaphyses (black arrow). E: Radiograph of the right humerus demonstrates slight irregularity of the distal humeral metaphysis with some central lucency (black arrow) as well as some generalized demineralization of the proximal humeral metaphysis. F: Radiograph of the right hand demonstrates mild demineralization of the metaphyses of multiple bones with some irregularity most prominent in the middle phalanges (black arrows). G: Radiograph of the right femur demonstrates irregularity of the distal right femoral metaphysis (black arrow). H: Radiograph of the right forearm demonstrates sclerosis and irregularity of the distal radial and ulnar metaphyses with some cupping of the distal ulnar metaphysis (black arrow). I: Radiograph of the left tibia and fibula demonstrate a celery stalk appearance of the distal metaphyses with coarsened trabecula, irregularity of the metaphysis and sclerosis (black arrow).

- The lower extremity radiographs demonstrated demineralization of the proximal and distal femoral metaphyses. Additional changes were noted in the bilateral calcanei. The upper extremity radiographs showed demineralization of the middle phalanges of the bilateral hands as well as the bilateral humeri. Irregularity, cupping and sclerosis of the bilateral distal radius and ulna were also seen (Fig. 3A–C).
- The neonate’s RPR test was nonreactive at birth and the rest of the workup was unremarkable.

### Triplet B

- The long bone radiographs showed demineralization of the hands (especially in the bases of the middle phalanges) and the proximal humeri. There was also central metaphyseal lucency of the distal humeri. The distal radius and ulna showed slight irregularity, sclerosis and cupping (Fig. 3D–F).
- The neonate’s RPR test was nonreactive at birth and the rest of the workup was unremarkable.

### Triplet C

- The infant was small for gestational age (a birthweight less than the 10th percentile for gestational age).
- The physical examination was significant for a diffuse papular erythematous rash of the chest, abdomen and extremities.
- The infant had leukopenia, neutropenia and thrombocytopenia. Bone imaging showed radiographic irregularities of the bilateral distal femoral metaphysis, left distal tibial metaphysis and celery stalk appearance of the left tibia and fibula. Additionally, the right forearm showed cupping of the distal ulnar metaphysis with some periosteal reaction (Fig. 3G–I).
- There was left-sided hearing loss on auditory brainstem response testing.
- The infant’s RPR test was nonreactive at birth and the rest of the workup was unremarkable.

## DISCUSSION

Bony involvement has been a long-observed feature of congenital syphilis.^13^ A prospective study by Brion et al^14^ highlights that abnormal long bone radiography in otherwise asymptomatic infants is an indication of active disease in newborns with congenital syphilis. Radiographic evidence of congenital syphilis was alarming and unexpected in both scenarios despite appropriately following treatment guidelines for maternal syphilis.^10^ Both mothers had RPR levels that were appropriately identified as serofast prior to conception and had adequate response to treatment (≥4-fold decrease in RPR titer) with no history indicating reinfection.

In case 1, the mother’s diagnosis and treatment for latent syphilis were complicated by discrepant RPR titers from separate labs. It is unclear from available records whether her final treatment was for reinfection or treatment failure. After the final dose of penicillin, she experienced a clear decrease in her RPR titer and met the established CDC criteria to be considered “serofast.” There was also no concern for reinfection following her most recent treatment. At delivery, her infant met the CDC criteria for congenital syphilis with a positive RPR test and radiographic evidence of congenital syphilis. The infant was subsequently treated with a 10-day course of penicillin G.

**FIGURE 4.**
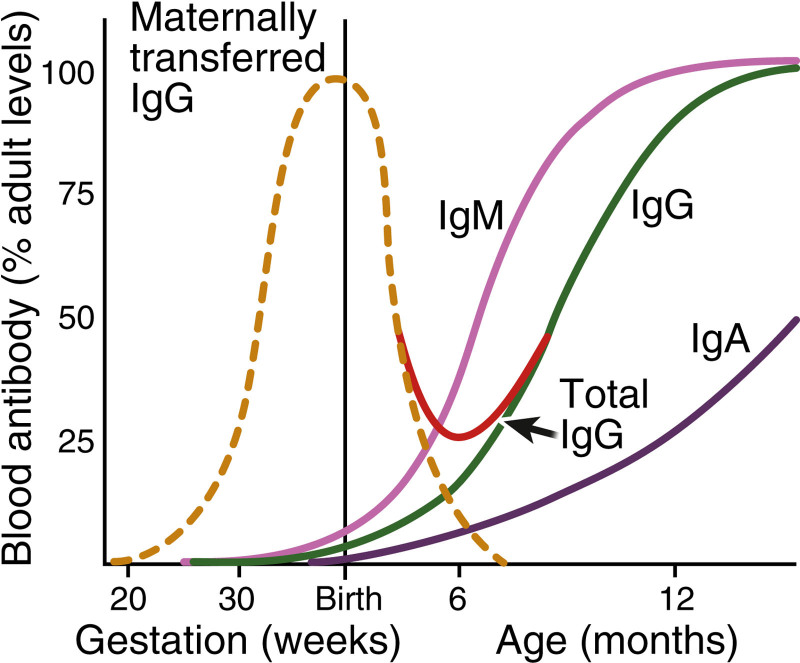
Neonatal immunoglobulin by age.

In case 2, all 3 infants had nonreactive RPR test results and the mother’s RPR titer was 1:2 near the time of delivery. This added some ambiguity to the diagnosis of congenital syphilis. Interestingly, a negative RPR result in an infant does not rule out congenital syphilis. Neonates, especially those born prematurely, have a relatively immature adaptive immune response, which likely explains the negative infant RPR in this setting. Even if positive, slightly elevated titers may represent maternal, rather than fetal nontreponemal titers. As Figure 4 demonstrates, premature infants are unlikely to produce their own antibody response to the LA at birth.^15^ This conclusion is also supported by findings from Chhabra et al^16^ who found that 14% of neonatal sera were RPR nonreactive in syphilitic women in the absence of treatment during pregnancy. A separate study of 348 pregnant women with positive serologies for syphilis found that 5% of cord blood samples were falsely negative for RPR with newborn physical findings consistent with congenital syphilis.^17^ Findings of a rash and thrombocytopenia consistent with congenital syphilis in 1 infant as well as long bone radiograph findings concerning for congenital syphilis in all 3 infants (read by 2 separate radiologists experienced in pediatric musculoskeletal radiology, with no alternative explanation for these findings) led to the diagnosis of congenital syphilis in these triplets. It is also likely that because these infants were fully treated with IV penicillin G for 14 days, they may never experience an elevated RPR titer in the future unless there is treatment failure or reinfection.

Current guidelines recommend that infants born to mothers with clearly documented appropriate treatment and serofast state do not need full evaluation for congenital syphilis unless clinically indicated. According to current testing algorithms, these infants would be classified as “congenital syphilis unlikely.”^3^ It remains unclear when and how often to retest individuals in a serofast state. Furthermore, retreatment is not routinely recommended for these individuals. Aside from the development of neurologic symptoms, there are also no clear guidelines to prompt a more in depth evaluation, such as CSF evaluation.^6^ These cases expose a vulnerability to our current syphilis guidelines and highlight the need for additional studies to understand the serofast state. In the 2022 report on sexually transmitted infections, the CDC reported that 12% of congenital syphilis cases were in patients whose mothers had been adequately treated.^18^ These examples reinforce that even in cases when congenital syphilis is deemed “unlikely,” the possibility of congenital syphilis remains.

It is possible that mothers with adequate treatment and a serofast LA titer are at risk for giving birth to infants with congenital syphilis. There is no gold standard test for diagnosing congenital syphilis. The diagnosis of congenital syphilis is determined based on clinical findings paired with appropriate laboratory and imaging data. This case series illustrates the lack of accurate and sensitive tests to detect congenital syphilis in such situations. Recent CDC laboratory recommendations for syphilis testing suggest that nucleic acid amplification testing may assist in the diagnosis of congenital syphilis.^6^ Yet, the actual role of nucleic acid amplification testing in congenital syphilis has yet to be established. Congenital syphilis diagnosis in the infants from this case series supports the need for improved diagnostics for mothers with a serofast state and their offspring.

Infants born to mothers with adequately treated and serofast status during the pregnancy should undergo a complete evaluation. Updates to current recommendations for the evaluation of congenital syphilis should be considered. We strongly advocate for close follow-up of these infants to monitor for treatment failure, as well as to coordinate various subspecialty care needs. Women with a serofast RPR test result who have given birth to newborns with congenital syphilis should be referred to an adult infectious disease specialist for a thorough history, physical examination and evaluation for optic, otic and/or neurosyphilis.

As we work together to address this global epidemic, we urge clinicians to carefully consider the possibility of congenital syphilis even in those infants who are born to mothers who had an adequate response to therapy but remain serofast.
